# Rheumatoid arthritis and interstitial lung disease: the role of comorbidities—a retrospective analysis of two RA inception cohorts in the UK

**DOI:** 10.1093/rheumatology/keag089

**Published:** 2026-02-16

**Authors:** Rositsa Dacheva, Amanda Busby, Patrick Kiely, Adam Young, David A Walsh, Daniel F McWilliams, James Galloway, Elena Nikiphorou

**Affiliations:** Independent researcher, Sofia, Bulgaria; School of Health, Medicine and Life Sciences, University of Hertfordshire, Hatfield, UK; Department of Rheumatology, St George’s University Hospitals NHS Foundation Trust, London, UK; School of Health, Medicine and Life Sciences, University of Hertfordshire, Hatfield, UK; Academic Rheumatology, University of Nottingham, Pain Centre Versus Arthritis, Nottingham, UK; Academic Rheumatology, University of Nottingham, Pain Centre Versus Arthritis, Nottingham, UK; Centre for Rheumatic Diseases, School of Immunology and Microbial Sciences, King’s College London, London, UK; Rheumatology Department, King’s College Hospital, London, UK; Centre for Rheumatic Diseases, School of Immunology and Microbial Sciences, King’s College London, London, UK; Rheumatology Department, King’s College Hospital, London, UK; Centre for Education, Faculty of Medicine and Life Sciences, King's College London, London, UK

**Keywords:** interstitial lung disease, rheumatoid arthritis, comorbidities, associated factors

## Abstract

**Objective:**

The objective of this study was to assess the role of comorbidities measurements in interstitial lung disease (ILD) in patients with RA.

**Methods:**

The data were from two consecutive multicentre, prospective RA inception cohorts, including baseline socio-demographic, clinical, treatment, laboratory and functional features. Comorbidity burden was assessed using a count of major comorbidities, the Rheumatic Disease Comorbidity Index (RDCI) and the Charlson Comorbidity Index (CCI). Associations between comorbidities and the development of ILD were explored, adjusted for clinical factors using multivariable logistic regression analyses.

**Results:**

The data from 2701 patients were included, and the median follow-up time was 6 years. In total, 101 patients (3.7%) were diagnosed with ILD. Twelve (0.44%) had the diagnosis at baseline, 46 were diagnosed during follow-up, and 43 were identified from death certificates. In multivariable analyses, age at onset [adjusted odds ratio (aOR) 1.03, 95% CI 1.01–1.05], seropositivity (aOR 2.58, 95% CI 1.38–4.81) and ever smoking (aOR 1.70, 95% CI 1.04–2.79) were associated with ILD diagnosis. Higher lung comorbidity burden measured using the lung disease component of the RDCI (aOR 4.59, 95% CI 2.68–7.88) was associated, as was using the entire index (aOR 1.32, 95% CI 1.07–1.63). The association did not remain when assessing comorbidity with alternative measures.

**Conclusion:**

The method chosen to assess the comorbidity burden affected whether baseline comorbidity was associated with subsequent ILD development. This study demonstrates that a specific Rheumatic Disease Comorbidity Index reveals associations not detected by generic tools or simple counts. Baseline lung comorbidities should be added to the known list of risk factors for ILD and aid targeted screening in the context of RA.

## Graphical abstract

**Graphical Abstract:**
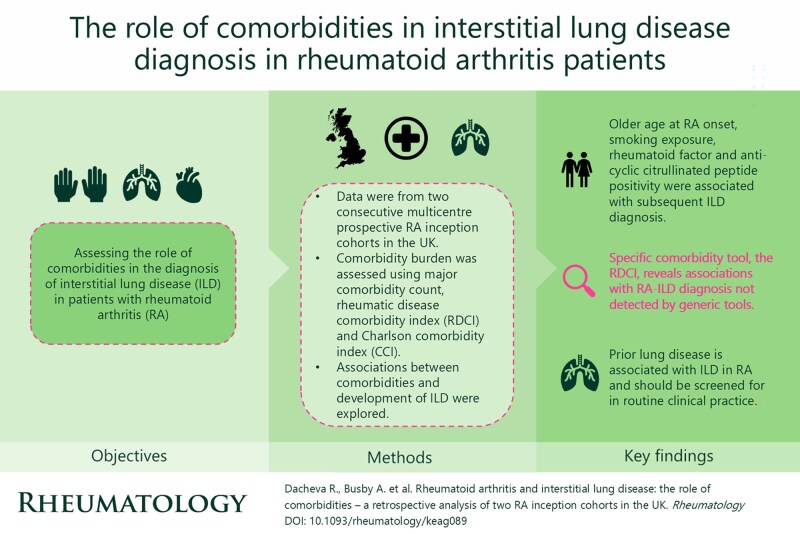

Rheumatology key messages

- The Rheumatic Disease Comorbidity Index reveals associations with RA-ILD diagnosis when more generic/simple comorbidity ‘tools’ may not.
- Prior lung disease is associated with ILD in RA and should be screened for in routine clinical practice.

## Introduction

RA is a chronic, inflammatory autoimmune disorder that primarily, but not exclusively, affects joints. Extra-articular manifestations occur in 40% of people with RA, the lungs being one of the most common non-articular sites of involvement [1]. Interstitial lung disease (ILD) remains the most strongly associated lung manifestation with RA and is a major contributor to morbidity and mortality in patients with RA [2, 3].

Pulmonary involvement may precede the onset of articular symptoms or can even be the presenting manifestation of disease [4]. The prevalence of RA-ILD varies widely, with estimates of 8% to 19%. This variation is largely driven by differences in sample sizes, study design, and other methodological aspects, as well as geographical variations [3, 5–8].

Previous reports revealed that up to 55% of people with RA have subclinical ILD noted on high-resolution CT (HRCT) [9, 10]. Reported risk factors for development of ILD include older age at disease onset, smoking exposure, and male sex [11, 12]. It is well known that smoking is a risk factor for RA onset, since tobacco exposure increases the risk of citrullination, and is associated with anti-CCP positivity [13, 14]. Moreover, anti-CCP positivity alone is strongly associated with incident RA-ILD [11].

Studies have previously assessed the association between comorbidity burden and RA-ILD using a variety of assessment methods. Hyldgaard *et al.* reported that comorbidity (measured using the Charlson Comorbidity Index, CCI) was higher in those with RA-ILD than matched RA people without ILD. Moreover, ischaemic heart disease, congestive heart failure, and diabetes were present more frequently in the RA-ILD group [7]. In contrast, previous work by our group found that the presence of major comorbidities at baseline (malignancies, cardiac disease, other non-cardiac cardiovascular conditions, diabetes, thyroid disease, OA, spinal disorders and gastrointestinal conditions) were associated with less RA-ILD [15] when adjusted for MTX use.

While the use of the CCI to reflect the comorbidity burden has been commonplace for some time, rheumatology-specific composite indices such as the Rheumatic Disease Comorbidity Index (RDCI) have been increasingly used in epidemiological studies [16]. Recognizing the unmet need to better understand the potential role of comorbidities in RA-ILD, specifically respiratory comorbidities, this study explored the impact of comorbidities on RA-ILD development. This is a retrospective analysis using data from two of the largest and longest follow-up RA inception cohorts in the UK.

## Methods

### Study population

The study included patients from two multicentre RA inception cohorts in the UK. The Early Rheumatoid Arthritis Study (ERAS) cohort included 1465 patients fulfilling the ARA criteria for RA recruited from 9 centres between 1986 and 2001 [14], maximum follow-up 25 years. Patients included in the ERAS cohort had <2 years symptom duration and were treatment naïve. The Early Rheumatoid Arthritis Network (ERAN) dataset included 1236 patients recruited 2002–2012 with disease duration of <3 years, maximum follow-up 10 years. All patients with a confirmed diagnosis fulfilling the ACR diagnostic criteria for RA were included in that study. Patients whose diagnosis changed to other non-RA diseases were excluded from these analyses.

### Independent variables

Socio-demographic, clinical, laboratory, and functional features were recorded for both cohorts at baseline, at 3–6 months, at 12 months, then once yearly as previously described [15, 17]. Assessments included clinical, laboratory and functional details; swollen and tender joint counts, HAQ disability index (HAQ-DI), ESR, RF, anti-CCP, BMI and plain film radiographs, the latter focusing on presence or absence of erosions. Serostatus was deemed positive if either RF or anti-CCP were detected. Smoking status was recorded at baseline in ERAN, but the ERAS baseline data was collected retrospectively at subsequent appointments.

The index of multiple deprivation (IMD) was calculated based on postcodes at baseline, grouped into quintiles. The date and main cause of death were retrieved from death certificates, provided by the National Health Service medical research information service (subsequently the NHS Health and Social Care Information Centre).

### Comorbidity measures

Three approaches, the RDCI, CCI and a major comorbidity count, were used to assess the impact of coexisting diseases at baseline. The presence of comorbid illnesses assessments were based on patient self-report, cross-checked by a rheumatologist.

The RDCI, specifically developed to evaluate the impact of comorbidities in patients with rheumatic diseases, has scores ranging from 0 to 9. It includes lung disease, cardiovascular disease, fracture, depression, diabetes mellitus, cancer, and ulcer or stomach problem [16], with the presence of lung disease and cardiovascular diseases being given twice the weight. Higher scores are indicative of a higher comorbidity burden. In addition, each subset of comorbidities was assessed independently. The RDCI is a validated tool specifically developed to assess the comorbidity burden in patients with rheumatic diseases and was included as a continuous variable in analyses.

The CCI is a general (non-RA specific) comorbidity index which takes into account 19 conditions, including chronic pulmonary diseases, each assigned a score of 1, 2, 3 or 6. These are summed and combined with points for age to produce a total score. Higher scores suggest higher predicted mortality rates [18].

Finally, major comorbidities were counted (‘simple count’), namely: malignancies, cardiac disease, non-cardiac cardiovascular conditions (e.g. hypertension, cerebrovascular disease), diabetes, thyroid disease, OA, spinal disorders, lung disease, and gastrointestinal conditions.

In addition, all three measures were assessed without the inclusion of lung disease to assess the potential impact of non-lung conditions alone, with ILD diagnosis.

### Interstitial lung disease

Baseline ILD cases were recorded for individuals with an existing diagnosis at study enrolment. Diagnosis of ILD during follow-up was based on relevant assessments, including imaging procedures (chest radiographs, HRCT scans, pulmonary function tests). Death certificates listing pulmonary fibrosis or ILD were used to ascertain diagnosis beyond the study follow-up, identifying individuals for whom ILD contributed to their death, either where a diagnosis was received prior to death (but beyond the study follow-up) or was determined post-mortem.

### Statistical analyses

The descriptive statistics summarized the baseline characteristics (Table 1). Odds ratios were estimated using logistic regression models to examine univariable associations between covariates and subsequent ILD diagnosis any time post-baseline (Supplementary Table S1). Multivariable logistic regression models were fitted, including a priori adjustments for age and gender. Other variables where *P* < 0.2 in univariable analysis were tested in multivariable analyses by comparing models using likelihood ratio tests (LRTs), retaining variables where LRTs yielded *P*-values of <0.05. Models were determined using complete cases, with multiple imputation (using chained equations) applied to account for missing smoking and serostatus data, once finalized. Individuals with baseline ILD were excluded from the analyses. Analyses were performed using Stata/MP v18.0.

**Table 1:** Baseline characteristics by ILD diagnosis.

		Interstitial lung disease diagnosed		No interstitial lung disease (*n* = 2600)
		Prior to RA diagnosis (*n* = 12)	Post RA diagnosis (*n* = 89)	
Age at RA onset (years), median (IQR)		68.5 (60, 73.5)	62 (56, 69)	57 (46, 67)
Female gender, *n* (%)		4 (33%)	48 (54%)	1756 (67.5%)
Minority ethnicity, *n* (%)		–	5 (6%)	77 (3.0%)
Index of Multiple Deprivation, *n* (%)				
1 (most deprived)		1 (10%)	14 (18%)	303 (14.0%)
2		1 (10%)	8 (10%)	365 (16.9%)
3		3 (30%)	29 (36%)	519 (24.1%)
4		5 (50%)	17 (21%)	443 (20.5%)
5 (least deprived)		–	12 (15%)	527 (24.4%)
Ever smoked, *n* (%)		8 (67%)	49 (68%)	1081 (52.8%)
Recruitment year, median (IQR)		1999 (1992.5, 2006.5)	1994 (1991, 2005)	1996 (1991, 2006)
BMI (kg/m^2^), median (IQR)		26 (22.7, 28.1)	25.8 (22.8, 29.4)	25.9 (23.1, 29.1)
HAQ-DI, median (IQR)		1.1 (0.6, 1.3)	1.3 (0.6, 1.9)	1 (0.5, 1.6)
DAS28, median (IQR)		4.8 (4.4, 6.3)	4.8 (4, 5.9)	4.7 (3.7, 5.7)
RF/anti-CCP positive, *n* (%)		7 (64%)	73 (84%)	1744 (71.7%)
Time to DMARD (months), median (IQR)		5 (3, 11)	6 (4, 11)	6 (4, 12)
**Rheumatic Diseases Comorbidity Index, *n* (%)**	0	–	51 (57%)	1825 (70.2%)
	1	–	11 (12%)	432 (16.6%)
	2	8 (67%)	20 (22%)	280 (10.8%)
	3	1 (8%)	4 (4%)	44 (1.7%)
	4	3 (25%)	3 (3%)	15 (0.6%)
	5	–	–	4 (0.2%)
Lung disease, *n* (%)		12 (100%)	20 (22%)	135 (5.2%)
Cardiovascular disease, *n* (%)		3 (25%)	8 (9%)	193 (7.4%)
Hypertension, *n* (%)		1 (8%)	9 (10%)	354 (13.6%)
Fractures, *n* (%)		–	–	7 (0.3%)
Depression, *n* (%)		–	1 (1%)	67 (2.6%)
Diabetes, *n* (%)		–	2 (2%)	52 (2.0%)
Ulcers/stomach diseases, *n* (%)		–	6 (7%)	73 (2.8%)
Cancer, *n* (%)		–	1 (1%)	71 (2.7%)
**Charlson Comorbidity Index, *n* (%)**	0	7 (58%)	68 (76%)	2167 (83.3%)
	1	5 (42%)	20 (22%)	319 (12.3%)
	2	–	–	94 (3.6%)
	3	–	1 (1%)	9 (0.3%)
	4	–	–	5 (0.2%)
	5	–	–	3 (0.1%)
	6	–	–	3 (0.1%)
**Count of major comorbidities, *n* (%)**	0	–	45 (51%)	1649 (63.4%)
	1	6 (50%)	35 (39%)	725 (27.9%)
	2	4 (33%)	7 (8%)	142 (5.5%)
	3	2 (17%)	1 (1%)	65 (2.5%)
	4	–	6	19 (0.7%)

IQR: interquartile range; HAQ-DI: health assessment questionnaire disability index; DAS28: 28-count DAS.

## Results

### ILD diagnoses

The analysis included data from 2701 patients; *n* = 101 (3.7%) received an ILD diagnosis at any time (*n* = 61 ERAS, *n* = 40 ERAN). Twelve of those (12%) had ILD prior to their RA diagnosis and were excluded from further analysis. During the study follow-up, 46/101 individuals (46%) were diagnosed with ILD. Forty-three (43%) were discovered to have ILD based on death certificate information post-study follow-up.

### Patient characteristics

Those with an ILD diagnosis after their RA diagnosis were older at RA onset (median 62 years, IQR 56–69) than those with no ILD (57 years, IQR 46–67, *P* < 0.001). There was a lower proportion of females in those with an ILD diagnosis (54%) than those without an ILD diagnosis (68%, *P* = 0.009) and more were current/past smokers (68% *vs* 53%, *P* = 0.010) or seropositive (86% *vs* 72%, *P* = 0.002). See Table 1 (baseline characteristics) and Supplementary Table S1 (univariable analyses).

Other variables associated with ILD diagnosis (where *P* < 0.2 in univariable analyses) were earlier recruitment year, more severe disability (higher HAQ-DI), seropositivity, and longer time to first DMARD, months). More had experienced previous RDCI-defined lung disease (22% *vs* 5%, *P* < 0.001). Additionally, a lower proportion of patients with ILD had no reported comorbidities compared with those without ILD, regardless of which comorbidity measure was used.

There were 167 individuals (6.2%) with lung disease at baseline, of whom 12 (7%) had baseline ILD and 20 (12%) subsequently developed it (Supplementary Table S2). One hundred and six had obstructive lung disease [asthma or chronic airway obstruction (COPD)] at baseline, four of whom (4%) later tested positive for ILD. Just 17 had baseline restrictive lung disease, 8 (47%) with ILD at baseline and the remaining 9 (53%) subsequently diagnosed.

### Multivariable analyses

The results from the multivariable analyses are shown in Table 2, with full models shown in Supplementary Table S3. Age at onset, smoking status, and seropositivity each increased the odds of subsequent ILD development, regardless of the comorbidity measure or whether any lung disease was included.

**Table 2:** Results of multivariable logistic regression analyses using multiple imputation (*n* = 2689).

	(A) Comorbidity, standard measure	(B) Comorbidity, excluding lung diseases	(C) RDCI, lung diseases only
	Odds ratio (95% CI)	Odds ratio (95% CI)	Odds ratio (95% CI)
Age at RA onset (years)^a^	1.03 (1.01, 1.05)*	1.04 (1.02, 1.06)*	1.03 (1.02, 1.05)*
Female gender^a^	0.70 (0.45, 1.08)	0.67 (0.43, 1.03)	0.70 (0.45, 1.10)
Ever smoked^a^	1.70 (1.04, 2.79)**	1.81 (1.10, 2.97)**	1.77 (1.03, 3.01)**
Seropositive^a^	2.58 (1.38, 4.81)*	2.59 (1.39, 4.83)*	2.54 (1.35, 4.78)*
Comorbidity			
Rheumatic Diseases Comorbidity Index (RDCI)	1.32 (1.07, 1.63)**	0.84 (0.61, 1.15)	4.59 (2.68, 7.88)*
Charlson Comorbidity Index (CCI)	0.87 (0.61, 1.25)	0.75 (0.50, 1.14)	–
Major comorbidity count	1.01 (0.78, 1.31)	0.94 (0.71, 1.24)	–

a Age at RA onset, gender, smoking status and seropositivity values shown from models which used RDCI to measure comorbidity. Similar values were obtained in models using CCI or major comorbidity count (see Supplementary Table S3). Results of seven models shown: (A) comorbidity measured with standard (i) RDCI, (ii) CCI and (iii) comorbidity count, (B) comorbidity measured with (i) RDCI, (ii) CCI and (iii) comorbidity count excluding lung diseases and (C) comorbidity measured with RDCI lung diseases only.

* *P* < 0.01.

** *P* < 0.05.

#### RDCI models

Each unit increase in RDCI score increased the odds of ILD [adjusted odds ratio (aOR) 1.32, 95% CI 1.07–1.63] in adjusted models. However, no association remained in analyses excluding all lung diseases from the comorbidity measure (aOR 0.84, 95% CI 0.61–1.15). Using only the lung disease component of RDCI (which includes all baseline lung diseases) significantly increased the adjusted odds of ILD (aOR 4.59, 95% CI 2.68–7.88).

#### CCI models

Neither of the CCI models demonstrated an association between comorbidities and ILD diagnosis (aOR 0.87, 95% CI 0.61–1.25 including any lung disease, aOR 0.75, 95% 0.50–1.14 excluding all lung diseases).

#### Count of major comorbidities models

Models using the count of major comorbidities also showed no association with ILD diagnosis, either when including (aOR 1.01, 95% CI 0.78–1.31) or excluding (aOR 0.94, 95% CI 0.71–1.24) all lung diseases.

**Figure 1:**
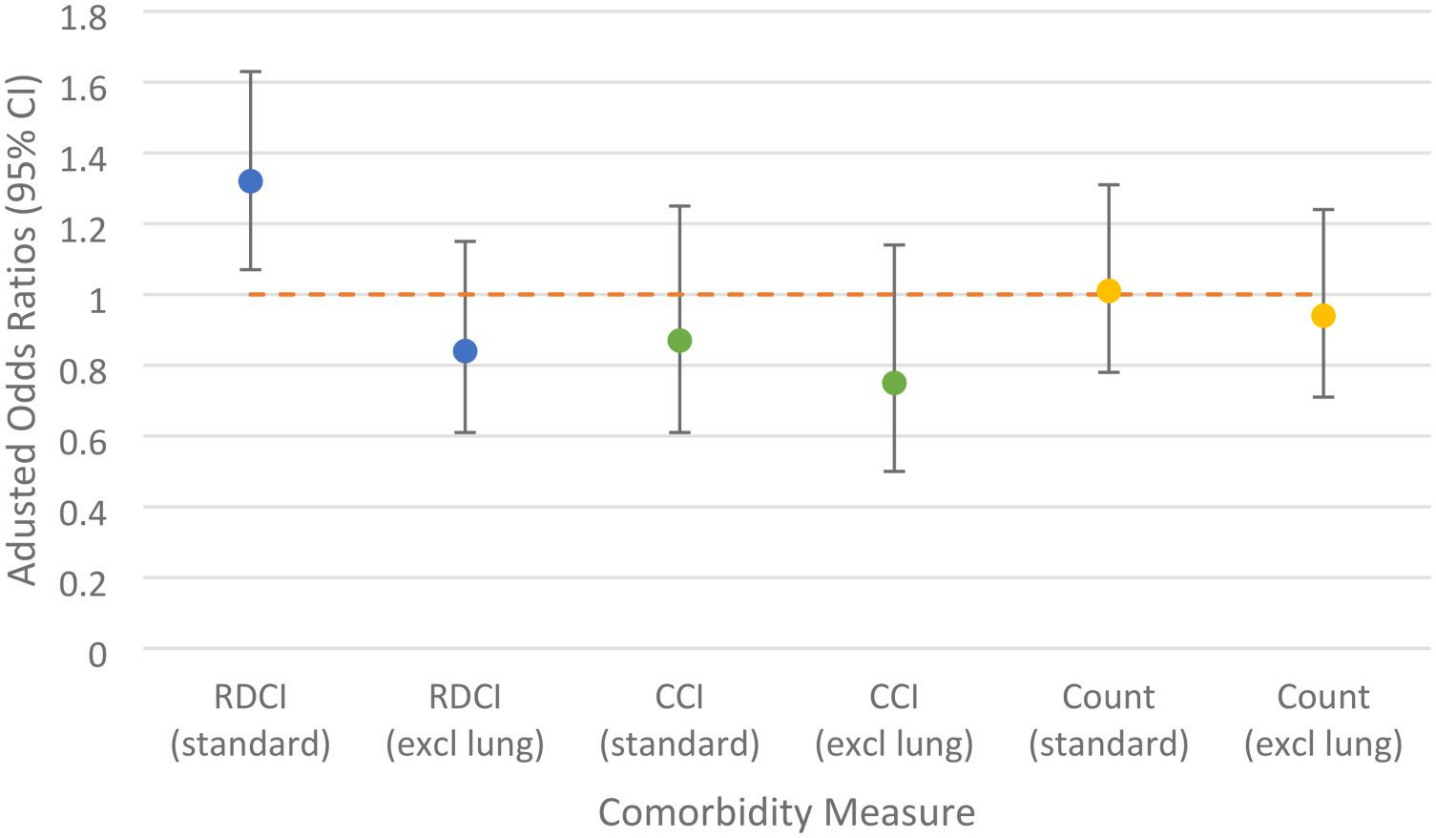
Comparison of adjusted odds ratios and 95% CIs of comorbidity measure used in each model. Odds ratio calculated in RDCI (lung only) excluded for clarity

A comparison of the aORs is shown in Fig. 1 for all models, excluding RDCI (lung diseases only), demonstrating that only the 95% CIs for the standard RDCI are above 1, indicating an association between RDCI and ILD diagnoses.

The results from the sensitivity analyses (Supplementary Table S4) were found to be consistent with those of the models shown, although with a reduction in smoking status ORs in all models. RDCI aORs were reduced in magnitude, though remained significant when using the entire index or only lung diseases. In complete-case analyses, without multiple imputation applied (Supplementary Table S5), the results remained consistent, although the observed association of RDCI with ILD diagnosis was reduced, with values below 1 included within the CI.

## Discussion

To assess the association between baseline comorbidity and related burden, with ILD development, comorbidity burden was measured in several ways. When considering the rheumatology-specific RDCI, comorbidity was found to be associated with ILD when using the standard index. However, this was not the case when all lung diseases were excluded, nor when measuring comorbidity using the CCI or the count of major comorbidities. This suggests that any predisposing lung disease may drive a higher risk for development of ILD. This finding suggests that ILD stratification in the clinical practice should focus on a history of prior lung diseases. Screening for lung diseases in patients with RA in the routine clinical practice may provide additional insights.

Comorbidities indisputably have an impact on treatment decisions and outcomes. Multimorbidity, defined as the coexistence of two or more chronic diseases in an individual, is common in patients with rheumatic diseases and often has implications on disease activity and treatment decisions, leading to less aggressive treatment. Although in ERAS and ERAN higher comorbidity burden measured with the RDCI was associated with increased odds of subsequent ILD development, this was not the case when prior lung disease was excluded. This aligns with previous work analysing this dataset, which found that RA-ILD diagnoses reduced with increased comorbidity burden (with prior lung disease excluded) when adjusted for MTX use. The underlying mechanisms are obscure, although a possibility of ascertainment bias, where those with certain morbidities, e.g. any other lung disease, are more likely to be investigated and therefore receive an ILD diagnosis, should be considered. Possible association between separate comorbid disorders and ILD might be caused by a common risk factor (such as smoking), or the comorbidities themselves might represent a distinct disease phenotype with extra-articular involvement. Undertreatment of patients with comorbidities due to concerns around polypharmacy, potential drug interactions and side effects, among other causes, may lead to ongoing disease activity and therefore ILD development. Biologic DMARDs have revolutionized the treatment of patients with RA. Mounting evidence indicates that early treatment and achieving disease remission may prevent the development of ILD by reducing systemic inflammation. This current work puts emphasis on the impact of lung comorbidities and their hypothetical role in subsequent ILD development. Addressing comorbidity burden at an early stage, the selection of an appropriate comorbidity index, and its correct interpretation is crucial in order to achieve better clinical outcomes.

This study supports previous observations of older age at disease onset and smoking exposure being associated with ILD [11, 15, 19]. Similarly, in line with previous studies, we found that RF and anti-CCP positivity were associated with subsequent ILD development [11, 12]. This report complements and adds to our previous work, highlighting baseline prevalence figures, the importance of the burden of lung comorbidity on the prediction of RA-ILD, and types of statistical modelling. From a methodological point of view, our study also demonstrates that the way comorbidities are measured can influence the results. Careful judgement on how to best utilize comorbidity data in epidemiological research, and caution with comparisons across studies, is therefore needed.

Our study has certain limitations, including the historical nature of the cohorts, with patients being recruited and followed up largely in the pre-biologics era. Although similar, the two cohorts used in these analyses were not contemporaneous, with some differences in the design, location and execution of the studies, as well as changes in standard treatment in line with best practice at the time. These differences may account for some of the variation seen in prevalence between the two cohorts. Furthermore, the diagnosis of ILD was not necessarily based on imaging criteria, which could result in an under-diagnosis. Another limitation is potential underestimation of true smoking status necessitating the imputation of missing values for smoking. Smoking status was collected after baseline in ERAS, so only collected for those still in study follow-up. However, by imputing we have likely underestimated the true smoking status (survivorship bias).

Nevertheless, the study has important strengths. These include the inception nature of the cohorts, the long follow-up, and the large number of patients recruited (pre-DMARD therapy). A further strength is the granular data available on various important clinical factors, particularly on comorbidities.

Given the complexity of ILD diagnosis and the significant morbidity caused, more epidemiological studies are needed to determine the prevalence of RA-ILD and to better understand associated risk factors, particularly the mechanism of association with other lung comorbidities, accepting that the prevalence may vary by geographical region and there can be management differences too. Recommendations on the screening of RA-ILD are generally lacking, perhaps because the absolute risks are too small to justify routine screening. Therefore, it is essential to identify people at risk for ILD development. The known clinical correlates of RA-ILD risk, also confirmed in our study are male sex, smoking, and seropositivity, and to these should be added any other baseline lung disease. A better understanding of ILD and its causes, will contribute to its timely diagnosis and optimal management in patients with RA, improving clinical outcomes and the overall prognosis.

In conclusion, older age at disease onset, smoking exposure, RF and anti-CCP positivity were associated with subsequent ILD development. Rheumatic-disease specific comorbidity indices, over other comorbidity tools, reveal association between comorbidity burden and RA with RA-ILD diagnosis. Prior lung disease is associated with ILD in RA and should be screened for in routine clinical practice.

The ERAS study received ethical approval from the West Hertfordshire Local Research Ethics Committee and subsequently from the Caldicott Guardian. The ERAN study received ethical approval from the Trent Research Ethics Committee.

## Supplementary Material

keag089_Supplementary_Data

## Data Availability

The data underlying this article will be shared on reasonable request to the corresponding author.
