# Supplementary material for: Rheumatoid arthritis and interstitial lung disease: the role of comorbidities—a retrospective analysis of two RA inception cohorts in the UK
Source: Rheumatology (Oxford). 2026 Feb 16;65(4):keag089. doi: 10.1093/rheumatology/keag089 (PMC13089416; doi:10.1093/rheumatology/keag089)
Supplement: keag089_Supplementary_Data [file keag089_supplementary_data.docx]

***Supplementary Table S1: Univariable analyses (those diagnosed with ILD in follow up or post-mortem v no ILD)***

|  |  |  | **Odds Ratio (95% CI)** | **p-value** |
| --- | --- | --- | --- | --- |
| ***Demographics*** | | |  |  |
|  | Age at RA onset *(years)* | | 1.03 (1.02, 1.05) | <0.001 |
|  | Female Gender | | 0.56 (0.37, 0.86) | 0.009 |
|  | Minority Ethnicity | | 1.94 (0.76, 4.91) | 0.201 |
|  | IMD quintile | | 0.91 (0.77, 1.07) | 0.243 |
|  | Ever Smoked | | 1.91 (1.15, 3.15) | 0.010 |
|  | Recruitment year | | 0.98 (0.95, 1.01) | 0.146 |
| ***Clinical*** | | |  |  |
|  | Body Mass Index *(kg/m^2^)* | | 0.99 (0.95, 1.04) | 0.706 |
|  | HAQ-DI | | 1.21 (0.92, 1.58) | 0.172 |
|  | DAS28 | | 1.09 (0.93, 1.27) | 0.279 |
|  | Seropositive | | 2.40 (1.29, 4.44) | 0.002 |
|  | Time to DMARD *(months)* | | 1.01 (1.00, 1.02) | 0.183 |
| ***Comorbidity measures*** | | |  |  |
|  | Rheumatic Diseases Comorbidity Index | | 1.51 (1.24, 1.83) | <0.001 |
|  |  | Lung disease | 5.29 (3.12, 8.96) | <0.001 |
|  |  | Cardiovascular disease | 1.23 (0.59, 2.58) | 0.591 |
|  |  | Hypertension | 0.71 (0.36, 1.43) | 0.323 |
|  |  | Fractures | - | - |
|  |  | Depression | 0.43 (0.06, 3.13) | 0.337 |
|  |  | Diabetes | 1.13 (0.27, 4.70) | 0.872 |
|  |  | Ulcers and other stomach diseases | 2.50 (1.06, 5.92) | 0.062 |
|  |  | Cancer | 0.40 (0.06, 2.95) | 0.299 |
|  | Charlson Comorbidity Index | | 1.08 (0.79, 1.48) | 0.655 |
|  | Count of major comorbidities | | 1.22 (0.96, 1.55) | 0.117 |
| ***Comorbidity measures (excl. lung disease)*** | | |  |  |
|  | Rheumatic Diseases Comorbidity Index | | 1.07 (0.80, 1.45) | 0.644 |
|  | Charlson Comorbidity Index | | 0.97 (0.67, 1.40) | 0.851 |
|  | Count of major comorbidities | | 1.14 (0.88, 1.47) | 0.337 |

*CI: confidence interval, IMD: Index of Multiple Deprivation, HAQ-DI: Health Assessment Questionnaire Disability Index, DAS28: Disease Activity Score 28 joint count, DMARD: disease modifying antirheumatic drugs.*

***Supplementary Table S2: interstitial lung disease status and lung disease type for those reporting lung disease at baseline (n=167)***

| **Lung Disease Type** | **No ILD** | **Baseline ILD** | **Subsequent ILD** | **Total** |
| --- | --- | --- | --- | --- |
|  | **N (%)** | **N (%)** | **N (%)** |  |
| Restrictive | 0 (0.0%) | 8 (47.1%) | 9 (52.9%) | 17 |
| Obstructive | 102 (96.2%) | 0 (0.0%) | 4 (3.8%) | 106 |
| Rheumatoid lung | 13 (61.9%) | 4 (19.0%) | 4 (19.0%) | 21 |
| Unknown | 20 (87.0%) | 0 (0.0%) | 3 (13.0%) | 23 |
| **Total** | **135 (80.8%)** | **12 (7.2%)** | **20 (12.0%)** | **167** |

*ILD: interstitial lung disease*

*Restrictive lung diseases include asbestosis, pneumoconiosis, pneumothorax, shrunken lung, pulmonary granuloma, post-inflammatory pulmonary fibrosis (idiopathic lung disease), pulmonary tuberculosis, sarcoidosis.*

*Obstructive lung diseases include asthma, bronchiectasis, chronic airway obstruction.*

*Rheumatoid lung diseases include pulmonary nodules, rheumatoid lung, pleural effusion.*

***Supplementary Table S3: Full Results of Multivariable Logistic Regression Analysis using Multiple Imputation (n=2,689)***

|  | **Rheumatic Diseases Comorbidity Index (RDCI)** | | | **Charlson Comorbidity Index (CCI)** | | **Major comorbidity count** | |
| --- | --- | --- | --- | --- | --- | --- | --- |
|  | **Standard measure**  **OR (95% CI)** | **Excluding lung diseases**  **OR (95% CI)** | **Lung disease only**  **OR (95% CI)** | **Standard measure**  **OR (95% CI)** | **Excluding lung diseases**  **OR (95% CI)** | **Standard measure**  **OR (95% CI)** | **Excluding lung diseases**  **OR (95% CI)** |
| Age at onset (years) | 1.03 (1.01, 1.05)** | 1.04 (1.02, 1.06)** | 1.03 (1.02, 1.05)** | 1.04 (1.02, 1.06)** | 1.04 (1.02, 1.06)** | 1.04 (1.02, 1.05)** | 1.04 (1.02, 1.06)** |
| Female Gender | 0.70 (0.45, 1.08) | 0.67 (0.43, 1.03) | 0.70 (0.45, 1.10) | 0.67 (0.43, 1.04) | 0.66 (0.43, 1.03) | 0.67 (0.43, 1.05) | 0.67 (0.43, 1.05) |
| Ever Smoked | 1.70 (1.04, 2.79)* | 1.81 (1.10, 2.97)* | 1.77 (1.03, 3.01)* | 1.80 (1.10, 2.95)* | 1.81 (1.11, 2.97)* | 1.77 (1.08, 2.90)* | 1.78 (1.09, 2.92)* |
| Seropositive | 2.58 (1.38, 4.81)** | 2.59 (1.39, 4.83)** | 2.54 (1.35, 4.78)** | 2.59 (1.39, 4.83)** | 2.60 (1.40, 4.84)** | 2.58 (1.39, 4.81)** | 2.58 (1.39, 4.81)** |
| Comorbidity measure | 1.32 (1.07, 1.63)* | 0.84 (0.61, 1.15) | 4.59 (2.68, 7.88)** | 0.87 (0.61, 1.25) | 0.75 (0.50, 1.14) | 1.01 (0.78, 1.31) | 0.94 (0.71, 1.24) |

***p<0.01, *p<0.05*

*OR: odds ratio, CI: confidence interval.*

***Supplementary Table S4: Results from sensitivity analyses, assuming those with missing smoker status were all smokers or all non-smokers (n=2,516)***

Results from sensitivity analysis using complete-case analyses, assuming a) all those with missing smoker status were smokers and b) all those with missing smoker status were non-smokers.

|  | **Rheumatic Diseases Comorbidity Index (RDCI)** | | | | | |
| --- | --- | --- | --- | --- | --- | --- |
|  | **Standard measure** | | **Excluding lung disease** | | **Lung disease only** | |
|  | **All Smokers**  **OR (95% CI)** | **Non-Smokers**  **OR (95% CI)** | **All Smokers**  **OR (95% CI)** | **Non-Smokers**  **OR (95% CI)** | **All Smokers**  **OR (95% CI)** | **Non-Smokers**  **OR (95% CI)** |
| Age at RA onset (years) | 1.03 (1.01, 1.05)** | 1.03 (1.01, 1.05)** | 1.04 (1.02, 1.06)** | 1.04 (1.02, 1.06)** | 1.03 (1.02, 1.05)** | 1.03 (1.02, 1.05)** |
| Female Gender | 0.69 (0.44, 1.08) | 0.69 (0.44, 1.08) | 0.66 (0.42, 1.03) | 0.67 (0.43, 1.04) | 0.70 (0.44, 1.09) | 0.70 (0.45, 1.10) |
| Ever Smoked | 1.47 (0.89, 2.41) | 1.45 (0.93, 2.27) | 1.49 (0.91, 2.44) | 1.59 (1.01, 2.48)* | 1.45 (0.88, 2.38) | 1.51 (0.97, 2.36) |
| Seropositive | 2.60 (1.39, 4.83)** | 2.59 (1.39, 4.82)** | 2.62 (1.41, 4.88)** | 2.59 (1.39, 4.82)** | 2.65 (1.42, 4.94)** | 2.62 (1.40, 4.89)** |
| Comorbidity measure | 1.26 (1.01, 1.57)* | 1.24 (0.99, 1.55) | 0.85 (0.61, 1.19) | 0.82 (0.59, 1.14) | 3.80 (2.15, 6.74)** | 3.83 (2.16, 6.79)** |

|  | **Charlson Comorbidity Index (CCI)** | | | |
| --- | --- | --- | --- | --- |
|  | **Including lung disease** | | **Excluding lung disease** | |
|  | **All Smokers**  **OR (95% CI)** | **Non-Smokers**  **OR (95% CI)** | **All Smokers**  **OR (95% CI)** | **Non-Smokers**  **OR (95% CI)** |
| Age at RA onset (years) | 1.04 (1.02, 1.06)** | 1.04 (1.02, 1.06)** | 1.04 (1.02, 1.06)** | 1.04 (1.02, 1.06)** |
| Female Gender | 0.66 (0.43, 1.04) | 0.67 (0.43, 1.05) | 0.66 (0.43, 1.04) | 0.67 (0.43, 1.04) |
| Ever Smoked | 1.49 (0.91, 2.44) | 1.54 (0.99, 2.41) | 1.49 (0.91, 2.44) | 1.56 (1.00, 2.44) |
| Seropositive | 2.61 (1.40, 4.86)** | 2.59 (1.39, 4.82)** | 2.61 (1.40, 4.86)** | 2.59 (1.39, 4.83)** |
| Comorbidity measure | 0.95 (0.67, 1.36) | 0.93 (0.65, 1.33) | 0.95 (0.67, 1.36) | 0.81 (0.53, 1.22) |

|  | **Major comorbidity count** | | | |
| --- | --- | --- | --- | --- |
|  | **Including lung disease** | | **Excluding lung disease** | |
|  | **All Smokers**  **OR (95% CI)** | **Non-Smokers**  **OR (95% CI)** | **All Smokers**  **OR (95% CI)** | **Non-Smokers**  **OR (95% CI)** |
| Age at RA onset (years) | 1.04 (1.02, 1.05)** | 1.04 (1.02, 1.06)** | 1.04 (1.02, 1.06)** | 1.04 (1.02, 1.06)** |
| Female Gender | 0.67 (0.43, 1.04) | 0.67 (0.43, 1.05) | 0.67 (0.43, 1.04) | 0.68 (0.43, 1.06) |
| Ever Smoked | 1.49 (0.91, 2.44) | 1.53 (0.98, 2.39) | 1.48 (0.90, 2.43) | 1.56 (0.99, 2.43) |
| Seropositive | 2.61 (1.40, 4.86)** | 2.58 (1.39, 4.81)** | 2.61 (1.40, 4.86)** | 2.58 (1.39, 4.81)** |
| Comorbidity measure | 1.02 (0.78, 1.34) | 0.98 (0.75, 1.30) | 0.94 (0.70, 1.25) | 0.90 (0.67, 1.21) |

***p<0.01, *p<0.05. OR: odds ratio, CI: confidence interval.*

***Supplementary Table S5: Results from complete-case analyses, without multiple imputation (n=1,957)***

|  | **Rheumatic Diseases Comorbidity Index (RDCI)** | | | **Charlson Comorbidity Index (CCI)** | | **Major comorbidity count** | |
| --- | --- | --- | --- | --- | --- | --- | --- |
|  | **Standard measure**  **OR (95% CI)** | **Excluding lung diseases**  **OR (95% CI)** | **Lung disease only**  **OR (95% CI)** | **Standard measure**  **OR (95% CI)** | **Excluding lung diseases**  **OR (95% CI)** | **Standard measure**  **OR (95% CI)** | **Excluding lung diseases**  **OR (95% CI)** |
| Age at RA onset *(years)* | 1.04 (1.02, 1.06)** | 1.04 (1.02, 1.06)** | 1.04 (1.02, 1.06)** | 1.04 (1.02, 1.06)** | 1.04 (1.02, 1.06)** | 1.04 (1.02, 1.06)** | 1.04 (1.02, 1.06)** |
| Female Gender | 0.72 (0.43, 1.19) | 0.70 (0.42, 1.16) | 0.72 (0.43, 1.19) | 0.70 (0.42, 1.16) | 0.70 (0.42, 1.16) | 0.70 (0.42, 1.16) | 0.70 (0.42, 1.16) |
| Ever Smoked | 1.90 (1.10, 3.27)* | 1.92 (1.12, 3.30)* | 1.92 (1.12, 3.30)* | 1.92 (1.12, 3.31)* | 1.93 (1.12, 3.32)* | 1.92 (1.11, 3.30)* | 1.91 (1.11, 3.29)* |
| Seropositive | 2.57 (1.26, 5.28)* | 2.58 (1.26, 5.29)* | 2.59 (1.26, 5.32)** | 2.58 (1.26, 5.30)* | 2.59 (1.26, 5.30)** | 2.58 (1.26, 5.28)* | 2.58 (1.26, 5.29)* |
| Comorbidity measure | 1.26 (0.96, 1.64) | 0.94 (0.65, 1.35) | 3.10 (1.55, 6.20)** | 0.93 (0.62, 1.40) | 0.90 (0.58, 1.37) | 1.02 (0.74, 1.41) | 0.96 (0.68, 1.35) |

***p<0.01, *p<0.05. OR: odds ratio, CI: confidence interval.*
